# Seasonal and daily variations in the occurence and outcomes of acute Stanford type A dissections: a retrospective single-center study

**DOI:** 10.1186/s13019-023-02222-4

**Published:** 2023-04-07

**Authors:** Qinmei Lin, Qingsong Wu, Xiaodong Chen, Xingfeng Chen, Linfeng Xie, Liangwan Chen

**Affiliations:** 1 Department of Outpatient, Union Hospital, Fujian Medical University, Fuzhou, Fujian People’s Republic of China; 2 Department of Cardiovascular Surgery, Union Hospital, Fujian Medical University, Xinquan Road 29, Fuzhou, Fujian People’s Republic of China; 3 Fujian Key Laboratory of Cardio-Thoracic Surgery (Fujian Medical University), Fuzhou, 350001 Fujian People’s Republic of China; 4 Fujian Provincial Special Reserve Talents Laboratory, Fuzhou, Fujian People’s Republic of China; 5 Fujian Medical University, Fuzhou, Fujian People’s Republic of China

**Keywords:** Weather conditions, South-eastern China, Stanford type A acute aortic dissection, In-hospital mortality

## Abstract

**Background:**

To investigate the seasonal, monthly, and daily distributions of the incidence of Stanford type-A acute aortic dissection (TAAAD) and identify seasonality in the duration of hospital stay and in-hospital mortality of TAAAD in south-eastern China.

**Methods:**

We enrolled patients diagnosed with TAAAD between 1 June 2017 and 31 May 2021. Participants were divided into seasonal, monthly, and daily groups according to the need for analysis. Analysis of variance was applied to compare the number of TAAAD in different seasons, months, and days. χ^2^ test was used to compare in-hospital mortality among the four groups. Non-parametric methods were used for all comparisons of the duration of hospital stay. Univariate logistic and multivariable logistic regression analyses were performed to assess the duration of hospital stay.

**Results:**

Of the 485 patients, 154 were diagnosed in winter (31.8%), 115 in spring (23.7%), 73 in summer (15.1%), and 143 in autumn (29.5%). The daily, monthly, and seasonal distributions of TAAAD were significantly different (*P* = 0.04, *P* < 0.01, and *P* < 0.01, respectively). This study did not identify any significant decrease in maximal, mean, or minimum temperatures between the three days before TAAAD and the day of TAAAD. No seasonal variations associated with in-hospital mortality was observed (*P* = 0.89). However, significant differences were observed in the seasonal distribution of the duration of hospital stay for TAAAD [winter was 17.0 (4.0–24.0) days, spring was 20.0 (14.0–29.0) days, summer was 20.0 (12.5–31.0) days, and autumn was 20.0 (13.0–30.0) days, *P* < 0.01]. Multiple factor analysis showed that winter was the independent risk factor for the increased duration of hospital stay. The odds ratio for winter was 2.21 (1.46, 3.33, *P* < 0.01).

**Conclusions:**

Our study confirmed that the incidence of TAAAD exhibits seasonal, monthly, and daily variations in south-eastern China. Moreover, the daily incidence of TAAAD is higher on weekdays than that on weekends.

**Supplementary Information:**

The online version contains supplementary material available at 10.1186/s13019-023-02222-4.

## Background

Type-A acute aortic dissection (AAD) (TAAAD) involves the ascending aorta, and is a life-threatening disease, with an incidence of approximately 2.0 cases per 100,000/year [1]. Moreover, Chinese patients with Stanford TAAAD are approximately 10 years younger than those in Europe and America [2, 3]. Therefore, identifying the risk factors that affect AAD is important for risk stratification. The inherent risk factors for the development of AAD, including age, sex, smoking, arterial hypertension, dyslipidaemia, and connective tissue disorders have been analysed [4]. Several studies have purported to demonstrate that cardiovascular diseases (CVD) exhibit distinct chronobiological rhythms, including daily, weekly, and seasonal cycles [5–7]. Many cardiovascular diseases, such as stroke [8–10], supraventricular tachycardia [11], coronary heart disease [12, 13], and heart failure [14], show peak admissions during the winter. While several publications have recorded the frequency of seasonal variations in AAD and showed that the incidence is lowest in summer and highest in winter [15–18], others have not observed any significant association with seasonality [6, 19, 20]. Because of the different patient populations, geographic locations, local climates, study designs, and statistical methodologies, we could not confirm the correlation between the incidence of TAAAD and weather conditions. Therefore, in this study, we aimed to evaluate the daily, monthly, and seasonal distributions of TAAAD and to further identify seasonality in the duration of hospital stay and in-hospital mortality of TAAAD in China. This retrospective study was conducted in south-eastern China.

## Patients and methods

### Study population

This study included all consecutive patients admitted to our department with a diagnosis of TAAAD between 1 June 2017 and 31 May 2021. We included patients who (1) came from Fujian province and nearby areas and (2) had a diagnosis of TAAAD, as confirmed by computed tomography angiography or magnetic resonance angiography. We excluded patients if (1) the exact date of TAAAD symptom onset was unknown, (2) they had a clear aetiology such as iatrogenic AD secondary to cardiac surgery, Loeys-Dietz syndrome, Marfan syndrome, or a history of surgery for AD, and (3) they had a history of other cardiac surgery or chronic aortic dissections. This retrospective study was approved by the Ethics Committee of our Hospital and was conducted in accordance with the Declaration of Helsinki. The Strengthening the Reporting of Observational studies in Epidemiology guidelines were followed in this retrospective study (Additional file 1).

Baseline characteristics included age, sex, date of onset, and medical history, including alcohol consumption status, smoking status, and hypertension. Other clinical records included baseline vital signs at admission (heart rate and systolic/diastolic blood pressure), laboratory inspections, and hospital admission (surgical intervention or medical therapy). We gathered data on in-hospital outcomes from medical records.

The patients in our study were divided into seasonal groups in south-eastern China, (spring: 1 March–30 May, summer: 1 June–31 August, autumn: 1 September–30 November, and winter: 1 December–28 February), monthly groups, or daily groups according to our need for analysis.

### Meteorological data

This study was conducted in Fuzhou (south-eastern China in Fujian province). This area has a subtropical monsoon climate. The mean daily temperatures range between 3 and 10 °C in winter and between 22 and 32 °C in summer. Daily regional meteorological data were retrieved from the Fuzhou Meteorological Service. The following parameters were assessed each day: minimum, mean, and maximum air temperatures (°C).

### Statistical analysis

Continuous variables were reported as mean±standard deviation (SD) or median and interquartile range (IQR) according to whether they followed a Gaussian distribution. Analysis of variance or χ^2^ test was used to compare baseline characteristics among the seasonal groups. χ^2^ test was used to analyse the differences in in-hospital mortality among the seasonal groups. Non-parametric methods were used for all comparisons of the duration of hospital stay. The cut-off point was considered according to the average duration of hospital stay of all patients, and variables with P<0.20 on univariate analysis were selected to perform multivariate logistic regression analysis on the related risk factors of patients, using generalized additive models to explore changes in weather and aortic dissection. The statistical significance of all tests was defined as *p* < 0.05 (two-sided). Statistical analyses were performed using SPSS V.22.0 and R software V.3.4.2. For individual cases of lost data, we used the mode principle of statistics to replace the missing values. The cases with several missing values (more than three) were omitted.

## Results

### Study population

**Fig. 1:**
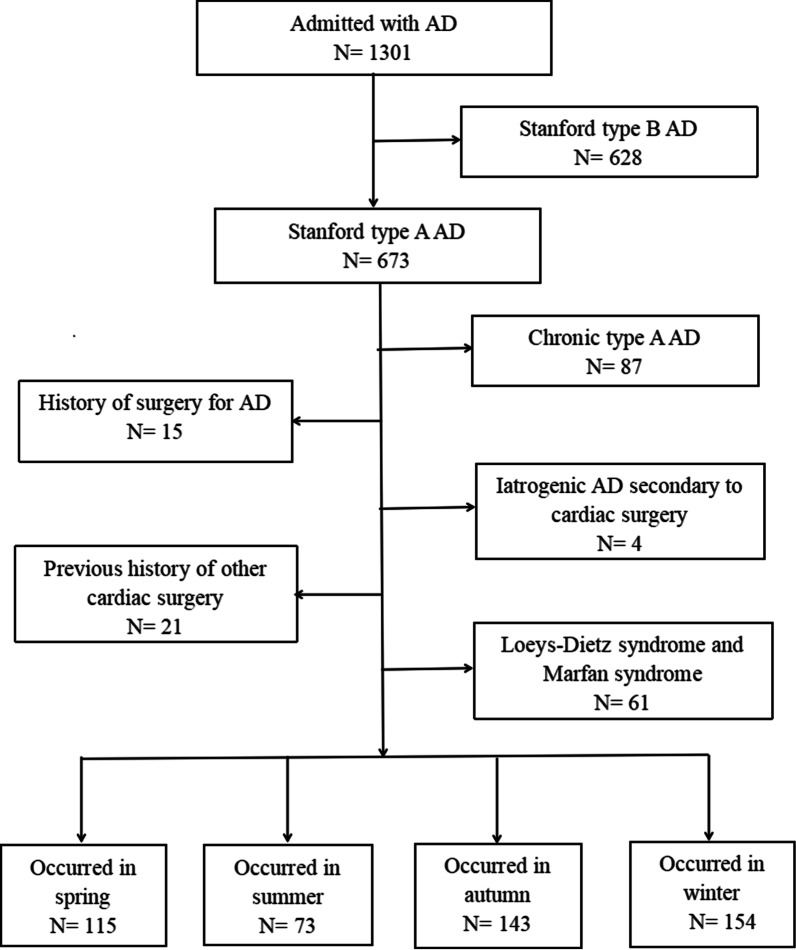
Consort type diagram of whole patients with AD

Between 1 June 2017 and 31 May 2021, 485 consecutive patients diagnosed with TAAAD in our department were enrolled in this study; of these, 154 cases of TAAAD occurred in winter (31.8%), 115 in spring (23.7%), 73 in summer (15.1%), and 143 in autumn (29. 5%) (Fig. 1). The study population comprised 365 (75.3%) men and 120 women with a mean age of 54.36 ± 11.94 months. The clinical characteristics of patients in the seasonal groups are summarized in Table 1. There were no differences in the distributions of most baseline characteristics among the seasonal groups. However, the number of patients with TAAAD that occurred in winter and were managed with surgical intervention were significantly lesser than those that occurred in the other seasons (*P *< 0.01).

**Table 1:** Baseline characteristics of patients with TAAAD onset in different seasons

Characteristic	Winter (N = 154)	Spring (N = 115)	Summer (N = 73)	Autumn (N = 143)	*P* value
Age, years	54.2 ± 12.4	56.2 ± 10.6	53.7 ± 11.2	53.3 ± 12.7	0.26
Male	113 (73.4)	84 (73.0)	60 (82.2)	108 (75.5)	0.48
Smoker	73 (47.4)	59 (51.3)	48 (65.8)	81 (56.6)	0.06
Alcohol consumption	70 (45.5)	58 (50.4)	44 (60.3)	74 (51.8)	0.22
SBP, mm Hg	139.3 ± 27.3	139.6 ± 28.3	136.4 ± 21.0	141.3 ± 32.0	0.69
DBP, mm Hg	77.1 ± 15.0	75.7 ± 15.0	77.0 ± 12.0	75.9 ± 18.9	0.85
Heart rate, beats/min	83.6 ± 16.0	83.1 ± 15.8	82.0 ± 16.4	84.5 ± 18.4	0.78
RBC count, × 10^12^ cells/L	4.1 (3.6–4.6)	4.2 (3.9–4.7)	4.2 (3.7–4.5)	4.2 (3.8–4.7)	0.31
Platelet count, × 10^9^ cells/L	158.0 (119.0–207.0)	170.0 (145.0–196.0)	158.0 (125.0–199.0)	170.5 (130.0–211.3)	0.21
Serum creatinine, μmol/L	92.0 (71.0–134.0)	85.0 (70.2–129.0)	93.0 (73.9–133.0)	88.1 (69.2–135.0)	0.63
Prothrombin time, s	13.9 (13.1–14.9)	13.7 (13.1–14.5)	13.9 (13.3–14.9)	14.2 (13.3–15.1)	0.13
Serum total protein, g/L	62.3 (57.7–66.9)	63.9 (60.0–67.2)	63.1 (59.9–67.9)	62.9 (57.7–67.2)	0.18
Serum albumin, g/L	34.8 (30.7–38.3)	35.5 (33.0–38.9)	35.6 (32.9–38.9)	35.5 (31.7–38.6)	0.24
Serum globulin, g/L	27.5 (24.3–30.5)	27.7 (24.5–30.3)	27.9 (25.4–30.8)	27.4 (24.5–30.1)	0.65
Surgical intervention	115 (74.7)	91 (79.1)	64 (87.7)	113 (79.0)	< 0.01

Values are mean ± SD, n (%) or median (IQR)

*AAD* Acute aortic dissection, *SBP* Systolic blood pressure, *DBP* Diastolic blood pressure, *RBC* Red blood cell

### Daily, monthly, and seasonal variations in symptom onset

**Fig. 2:**
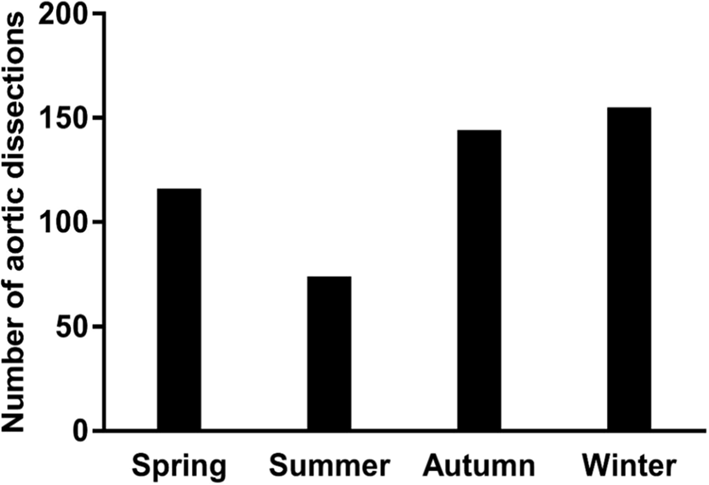
Seasonal distribution of AAD. AAD: acute aortic dissection

**Fig. 3:**
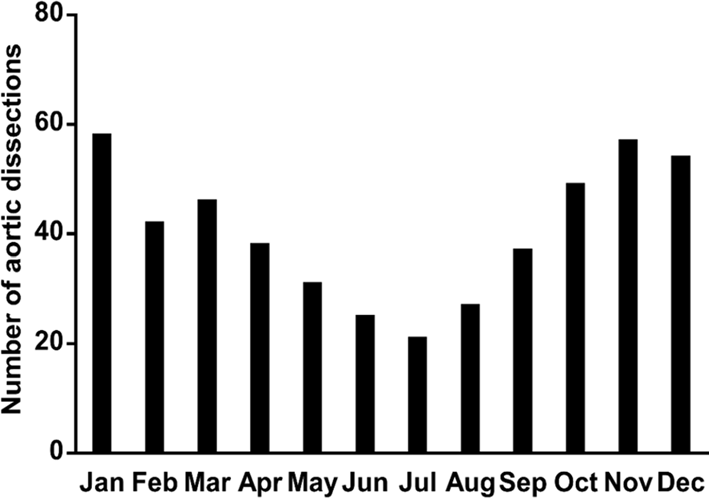
Monthly distribution of AAD

**Fig. 4:**
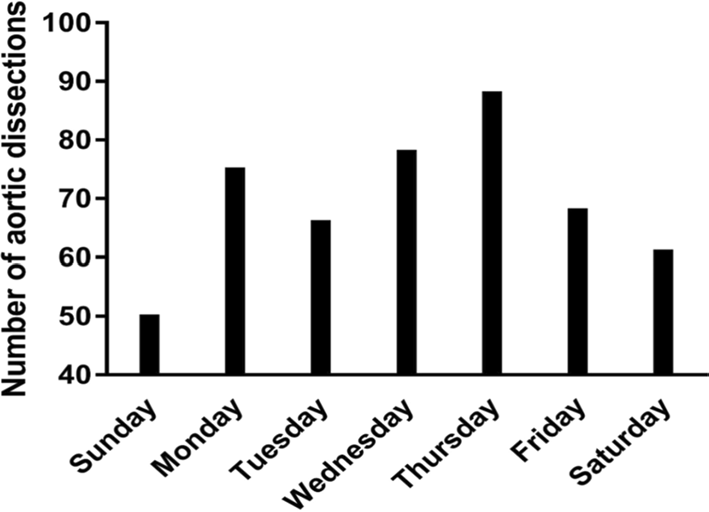
Daily distribution of AAD

The daily, monthly, and seasonal variations in the onset of TAAAD symptoms are presented in Figs. 2, 3, and 4, respectively.

**Fig. 5:**
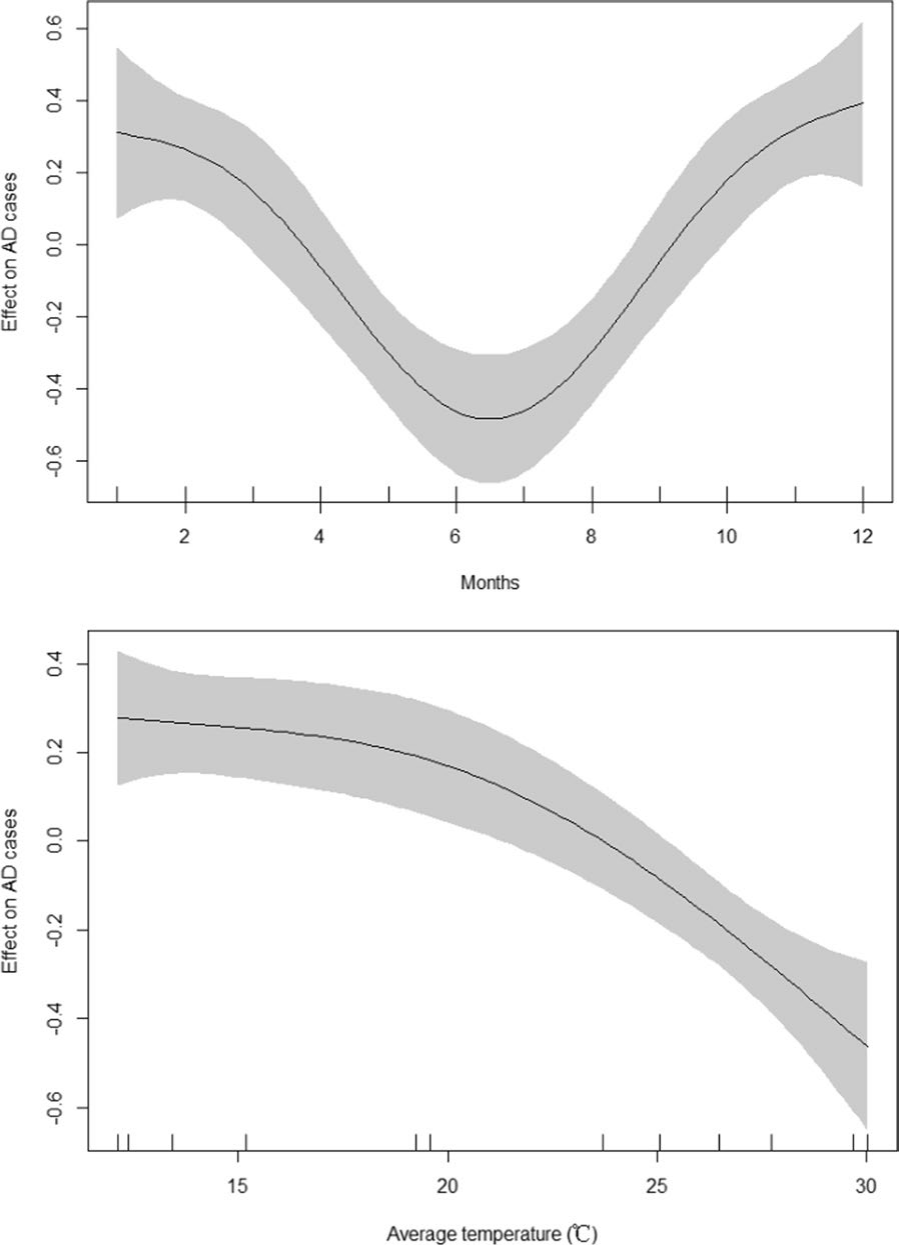
Generalized additive models for months and average temperature effect on TAAAD cases

Significant variations in TAAAD symptom onset were investigated; the onset of TAAAD was 2.11 times higher in winter than that in summer (*P* < 0.01). Moreover, the frequency of TAAAD in January was three times higher than that in July (*P* < 0.01), and the daily distribution in TAAAD was significantly different (*P* = 0.04). The frequency of TAAAD was higher on weekdays than that on weekends (*P* < 0.01). The generalized additive models showed that months and average temperature have a non-linear relationship with the incidence of TAAAD. The lower the average temperature, the more the cases of TAAAD. Similarly, the colder the months (winter), the more the cases of TAAAD [R-sq.(adj) = 0.91, deviance explained = 94.7%], as presented in Fig. 5. The onset of TAAAD was 1.50 times higher on Mondays than that on Sundays. For example, the frequency of TAAAD on Thursday was 1.44 times higher than that on Saturday and 1.76 times higher than that on Sunday.

### Weather variations during the days preceding TAAAD

The variations in atmospheric temperatures during the three days preceding TAAAD are presented in Table 2. For each patient, the maximal, mean, and minimal atmospheric temperatures on the AAD day were obtained from the Fuzhou Meteorological Service and compared with data from the three preceding days. Our study did not observe any significant differences in the decrease in maximal, mean, and minimal temperatures between the days before and after AAD.

**Table 2:** Atmospheric temperature variations during the days preceding AAD

Atmospheric temperature	D-3	D-2	D-1	AAD day
Minimal temperature (°C)	16.49 ± 6.72 *P* = 0.63	16.30 ± 6.75 *P* = 0.96	16.27 ± 6.71 *P* = 0.98	16.28 ± 6.69
Mean temperature (°C)	19.89 ± 6.86 *P* = 0.68	19.73 ± 6.95 *P* = 0.96	19.70 ± 6.88 *P* = 0.98	19.71 ± 6.85
Maximal temperature (°C)	23.30 ± 7.21 *P* = 0.73	23.16 ± 7.37 *P* = 0.97	23.15 ± 7.27 *P* = 0.98	23.14 ± 7.21

Results are presented as mean ± S.D

Bold values: *P* < 0.05

*AAD* Acute aortic dissection, *SD* Standard deviation

### In-hospital mortality and duration of hospital stay in patients with TAAAD onset in different seasons

The in-hospital mortality and duration of hospital stay in patients with TAAAD onset in different seasons are presented in Tables 3 and 4, respectively. In our study, the distribution of in-hospital mortality in patients diagnosed with TAAAD did not display seasonal variations (*P *= 0.89). However, the seasonal distribution of the duration of hospital stay for TAAAD revealed significant differences (*P *< 0.01). The duration of hospital stay for TAAAD was higher in winter than that in spring (*P *= 0.04) and autumn (*P *= 0.03). Univariate analysis of the duration of hospital stay showed that winter, autumn, coronary malperfusion, cerebral malperfusion, acute renal failure, acute aortic regurgitation, and serum creatinine are risk factors for the increased duration of hospital stay. Multiple factor analysis showed that winter was the independent risk factor for the increased duration of hospital stay. The odds ratio of winter was 2.21 (1.46, 3.33), *P *< 0.01 (Table 5).

**Table 3:** In-hospital mortality and the duration of hospital stay in patients with TAAAD onset in different seasons

	Winter	Spring	Summer	Autumn	*P* value
In-hospital mortality	12.3%	10.4%	9.6%	9.8%	0.89
The duration of hospital stay (days)	17.0 (4.0–24.0)	20.0 (14.0–29.0)	20.0 (12.5–31.0)	20.0 (13.0–30.0)	< 0.01

Values are presented as percentage or median (IQR)

Bold values: *P* < 0.05

**Table 4:** Multiple comparison of the duration of hospital stay in patients with TAAAD onset among different seasons

*P* value	A versus B	A versus C	A versus D	B versus C	B versus D	C versus D
The duration of hospital stay	0.031	0.095	0.043	1.000	1.000	1.000

*A* Winter, *B* Spring, *C* Summer, *D* Autumn

Bold values: *P* < 0.05

**Table 5:** Univariate and multivariate logistic regression analyses of risk factors for the duration of hospital stay

Valuables	Univariate		Multivariate	
	*P*	OR (95%CI)	*P*	OR (95%CI)
*Preoperative factors*				
Age	0.46	0.99 (0.98, 1.00)	–	–
Male	0.35	1.2 (0.81, 1.86)	–	–
Hypertension	0.56	0.88 (0.57, 1.36)	–	–
Diabetes mellitus	0.33	1.58 (0.63, 4.00)	–	–
Coronary heart disease	0.21	1.69 (0.75, 3.81)	–	–
*Preoperative complications*				
Coronary malperfusion	0.11	3.58 (0.77, 16.75)	0.07	4.71 (0.91, 24.51)
Cerebral malperfusion	0.11	0.33 (0.08, 1.27)	0.14	0.35 (0.09, 1.41)
Extremity malperfusion	0.72	0.77 (0.19, 3.13)	–	–
Acute renal failure	0.10	0.53 (0.25, 1.13)	0.38	0.70 (0.31, 1.56)
Acute aortic regurgitation	0.14	1.40 (0.90, 2.17)	0.34	1.25 (0.79, 1.98)
Serum creatinine	0.11	1.00 (0.99, 1.00)	0.07	1.00 (0.99, 1.00)
Serum albumin	0.22	0.97 (0.93,1.02)	–	–
RBC count	0.66	1.00 (0.99, 1.01)	–	–
Platelet count	0.22	1.00 (1.00, 1.01)	–	–
Winter	< 0.01	2.17 (1.45, 3.25)	< 0.01	2.21 (1.46, 3.33)
Spring	0.42	0.84 (0.55, 1.28)	–	–
Summer	0.42	0.84 (0.55,1.28)	–	–
Autumn	0.09	0.71 (0.48, 1.05)	0.87	0.96 (0.62, 1.49)

Those factors *p* < 0.200 in univariate model were involved in multivariate model

Bold values: *P* < 0.05

*RBC* Red blood cell

## Discussion

Our findings confirm that the incidence of TAAAD varies seasonally, monthly, and daily in south-eastern China. In this study, the occurrence of TAAAD was independent of the rapid change in atmospheric temperature. Moreover, the distribution of in-hospital mortality in patients with TAAAD did not show seasonal variations. However, the seasonal distribution of the duration of hospital stay for TAAAD was inhomogeneous in this study.

A series of studies showed that the fall and winter months represent a high-risk temporal frame for the occurrence of aortic dissections [9, 15, 17, 21, 22], which is consistent with our findings. A meta-analysis that included 60,567 cases of acute aortic rupture and dissection (AARD) suggested that the occurrence of AARD increases in winter [23]. Our study indicated that TAAAD incidence was lowest in July and highest in January, which is consistent with the results of an American study of 89,365 patients [17]. In addition, a study of 4615 patients with AAD in Italy suggested that the lowest incidence of AAD was in August, and the highest incidence was in January [22], although the reason for this seasonality remains unclear. However, increased sympathetic activity, high blood pressure [24], or changes in haematological properties may enhance the forces that produce considerable deformation and increase friction and shear stress on the internal surface [25], which makes the aorta vulnerable to high blood pressure [26]. This may be another relevant factor in the seasonal pattern of TAAAD.

In the present study, we found a distribution of the daily incidence of AAD, with a significantly higher incidence on weekdays than that on weekends. This finding is similar to that reported by Karangelis et al. [7]. A similar result was also observed in a study by Manfredini [27]. According to these findings, emotional and occupational stress may precipitate transient severe hypertensive responses and could be a risk factor of AAD, which deserves further investigation. During the entire work week, active or high-stress jobs coupled with a frenetic career pace have a major impact on our health; in comparison, weekends provide leisure and rest. This is in accordance with the frequency of TAAAD, which was higher on weekdays than that on weekends.

Benouaich et al. suggested that the relative change in temperature, rather than absolute temperature, is a mechanistic factor of TAAAD [28]. However, Karangelis et al. [7] found that the occurrence of AAD was not associated with rapid alterations in atmospheric temperature. Our study revealed no significant differences in the comparison of temperature on the day of TAAAD and on the three preceding days. In this study, meteorological data were retrieved from the local meteorological service to ensure that the results were relevant. It is possible that the data on the temperature of the three preceding days was insufficient, and further study is needed to collect more data on the preceding days to clarify this point. Another explanation for our finding may be that rapid alterations in atmospheric temperature in the Fujian region are not very common.

Previous studies investigated whether the onset season acted as a trigger in the prognostic value of patients with AAD; however, no association with seasonal variability could be found on the patients’ outcome and in-hospital mortality [17, 29]. In our study, we also did not observe any association with seasonality on in-hospital mortality, which is different from a study conducted in Beijing, located in northern China [21]. This inconsistency may be explained by the different seasonal patterns in Beijing and Fujian. Northern China has distinct seasonal patterns characterized by the transition from extremely hot wet summers to extremely cold dry winters during a short period in the first month of autumn [30, 31]. However, Fuzhou has a typical subtropical monsoon climate, with suitable temperatures, such as sunny, warm, humid, evergreen, abundant rainfall, less snow, and less frost. The annual average temperature is 20–25 °C.

Our analysis is the first to suggest that the duration of hospital stay is affected by the season of disease onset. Compared with spring and autumn, the duration of hospital stay in winter was the lowest. The in-hospital mortality was the highest in winter, and many patients were in serious condition and were extremely late to be cured; this is in accordance with the duration of hospital stay in winter, which was the lowest. However, there was no significant difference in the duration of hospital stay between winter and summer. There is a possibility that the number of hospitalized patients in summer is relatively less compared to that during other seasons, meaning that the duration of hospital stay is lesser. Further studies are needed to identify the factors that affect seasonal changes in hospitalization duration.

### Limitations

This study has some important limitations. A major limitation of our study is that our meteorological data did not include atmospheric pressure, humidity, and other specific climate data, which may provide more details regarding the reason for seasonal variability. Second, the number of preceding days that were investigated may be insufficient, meaning that the influence of rapid alterations in atmospheric temperature on TAAAD were not accurately investigated. Third, our study had a single-centre design in one province, and the sample size of this study was small. Additionally, the findings might not necessarily be reflected in other countries or cities with different climates. China has a large land area; thus, follow-up studies on the differences between the south and north and the east and west, and a multi-centre study are our targets. At present, the research period is four years, which may lead to some bias in the research results.

## Conclusion

Our study confirmed that the incidence of TAAAD exhibits seasonal, monthly, and daily variations in south-eastern China. Moreover, the daily incidence of TAAAD is higher on weekdays than that on weekends. This could promote the formulation of effective prevention strategies during the colder months, and further studies should seek to clarify the pathophysiology of seasonality in the incidence of TAAAD.

## Supplementary Information

**Additional file 1.** STROBE Guidelines.

## Data Availability

All data generated or analysed during this study are included in this published article.

## Abbreviations

AD: Aortic dissection
AAD: Acute aortic dissection
TAAAD: Stanford type-A acute aortic dissection
CVD: Cardiovascular diseases
SD: Standard deviation
IQR: Interquartile range
AARD: Acute aortic rupture and dissection
